# Correlation between echocardiographic severity of ischemic mitral valve regurgitation following acute myocardial infarction and its electrocardiographic location 

**DOI:** 10.15171/jcvtr.2018.27

**Published:** 2018-09-24

**Authors:** Mehrnoush Toufan, Sakineh Hadi, Afshin Habibzadeh

**Affiliations:** ^1^Cardiovascular Research Center, Tabriz University of Medical Sciences, Tabriz, Iran; ^2^Department of Internal Medicine, Ardebil University of Medical Sciences, Ardebil, Iran

**Keywords:** Myocardial Infarction, Mitral Valve Insufficiency, Mitral Regurgitation, Echocardiography

## Abstract

***Introduction:*** Ischemic mitral regurgitation (IMR) is common after acute myocardial infarction (AMI) which is associated with long-term cardiovascular mortality. Size, transmurality and location of the myocardial infarction (MI) has role on the development of IMR. In this study we evaluated the severity of IMR after different types of MI.

***Methods:*** One-hundred patients with the first AMI were recruited and according to echocardiographic findings were categorized to have moderate to severe IMR (case group, n=50) or trivial or no IMR (control group, n=50). Demographic and echocardiographic findings and MI location were compared between groups.

***Results:*** Case group compared to control group had significantly higher Killip class, more cases with left ventricular ejection fraction (LVEF) <30% and inferolateral STEMI. They had significantly higher left ventricular (LV) and right ventricular (RV) diastolic dysfunction. Mechanism of IMR was mono leaflet tethering in 88%, both leaflets tethering in 12% and ring dilatation in 62%. MR jet origin-direction was medial commisure-posterior in 66%, lateral commisure-anterior in 11 22% and both commisure-central direction in 12%.

***Conclusion:*** IMR is common after AMI, especially in cases with inferior MI. The echocardiographic findings are indicative of left ventricular remodeling and abnormality of mitral valve apparatus.

## Introduction

Myocardial infarction (MI) accompanies with different mechanical complications including ischemic mitral regurgitation (IMR) which is correlated with increased risk of mortality and heart failure.^1,2^ The prevalence of IMR after MI is estimated to be up to 50%.^3^

Echocardiography is the choice imaging modality for the diagnosis and assessment of IMR and establishing its etiology.^4^ IMR usually results from papillary muscle displacement, ventricular dilation and remodeling with reduced closing forces.^5^

Studies have shown that the incidence of IMR in inferior MI is higher than other types, but the outcome is worse for anterior MI.^6^ IMR existence and its severity had significant prognostic effect on patients’ mortality following AMI. We evaluated the prevalence of IMR and its severity in patients with AMI and its association with the infarct location and clinical condition.

## Materials and Methods

One hundred patients over 18 years with first time AMI including 50 patients with moderate to severe IMR (case group) and 50 patients with mild or no IMR (control group) admitted to Shahid Madani hospital during 2011-2012 were recruited. Exclusion criteria was previous MI and percutaneous transluminal coronary angioplasty (PTCA), performing PTCA before echocardiography during the admission, previous coronary artery bypass surgery (CABG), history of chordae or papillary muscle rupture, valvular or congenital heart disease.

Baseline demographic, clinical and procedural data was recorded for each patient; also, the infarction size and type of AMI (STEMI or NSTEMI and anterior vs inferior STEMI) were recorded according to ECG findings.

All patients underwent transthoracic echocardiography at rest using Vivid 7 cardiovascular ultrasound system (GE Healthcare, Little Chalfont, UK) during the first 5 days after AMI. Echocardiographic findings including left ventricular ejection fraction (LVEF), regional wall motion abnormality (RWMA), wall motion score index (WMSI), mitral inflow, left atrium, left ventricle and right ventricle sized were measured.

When the valve structure intrinsically were normal and the regurgitation was caused as a result of the STEMI, the ischemic MR was diagnosed. Control group had no IMR, trivial IMR or mild (MR<2+) and cases had moderate or severe MR (MR≥2+).

Modified biplane Simpson method was used to measure LV volume. LVEF was calculated from the LV end-diastolic and end-systolic volumes. WMSI was measured adding and dividing scores of regional wall motion of the all 17 LV segments. From the end systolic apical 4-chamber view, the LV sphericity was calculated by using the LV short-axis/long-axis dimension ratio. The prolate ellipsoid model was used to assess LAVI. Diastolic early filling velocity (E) wave, late diastolic velocity (A) wave, E/A ratio and DCT were measured via pulsed wave Doppler mitral inflow velocities. A restrictive LV filling pattern was defined as an E/A ratio >2, with a deceleration time of <150 ms. Also myocardial systolic (Sa), early diastolic (Ea), late diastolic (Aa) velocities, and E/E’ ratio were measured and averaged via pulsed wave TDI after placement of the sample volume at the level of the lateral and septal mitral annuli.^7^ Degree and severity of MR was assessed semi quantitatively and quantitatively using the proximal isovelocity surface area method, effective regurgitant orifice area, jet eccentricity, and jet area to atrial area, also measured the vena contracta width. The regurgitant volume, fraction, and orifice area were calculated via the volumetric or the proximal isovelocity surface area method.^4^

Measuring diastolic function and defining the grade of diastolic dysfunction according to conventional grading in MR is complicated due to the distortion in E and A velocities and E/A ratio. In these cases, other echocardiographic data such as DCT and findings of tissue Doppler (E/E’ ratio) also were used to evaluate the diastolic function.

### Statistical analysis

All data were analyzed using SPSS 15 software. The results are expressed as Mean ± standard deviation (SD) or percentage. Chi-square test, Fischer exact test and independent *t* test were used to compare data between groups. *P* values <0.05 were considered statistically significant.

## Results

Patients’ baseline findings between groups are shown in Table 1. Case group was significantly more women, older and smoker with higher previous stable and unstable angina with less streptokinase treatment, and with significantly higher Killip class, cases with LVEF<30% and inferolateral STEMI. There were only 5 cases (10%) of NSTEMI in case group.

**Table 1:** Baseline Findings between groups

	**Case group**	**Control Group**	***P*** ** value**
Age	64.6±10.7	59.9±11.7	0.03
Gender			
Male	26 (52%)	40 (80%)	0.003
Female	24 (48%)	10 (20%)	
Diabetes Mellitus	12 (24%)	5 (10%)	0.06
Hypertension	28 (56%)	20 (40%)	0.1
Hyperlipidemia	10 (20%)	10 (20%)	-
Smoking	10 (20%)	20 (40%)	0.02
Previous stable angina	13 (26%)	5 (10%)	0.03
Previous unstable angina	14 (28%)	3 (6%)	0.003
Previous exertional dyspnea	7 (14%)	3 (6%)	0.18
Normal chest X-ray	11 (22%)	35 (70%)	<0.001
MI type in ECG			
Anterior	10 (20%)	27 (54%)	0.003
Inferolateral	35 (70%)	23 (46%)	
LVEF			
<30%	18 (36%)	5 (10%)	0.001
30-45%	22 (44%)	21 (42%)	
>45%	10 (20%)	24 (48%)	
Killip class			
I	17 (34%)	44 (88%)	<0.001
II-III	33 (66%)	6 (12%)	
Treatment with streptokinase	28 (56%)	39 (78%)	0.019

Case group had significantly higher LVESV, LVESVI, LVEDV, LVEDVI, WMSI, LAD, MVEV, MV annulus size and index, inter-papillary muscle distance (systolic) and PAP and lower MVDCT, MV E/A, MV annulus excursion, inter-papillary muscle distance ratio and TV excursion (Table 2). Tissue Doppler findings were also significantly different between groups.

**Table 2:** Echocardiography and tissue Doppler findings between groups

	**Case group**	**Control Group**	***P*** ** value**
LVESV (mL)	80.7±40.00	54.2±27.9	<0.001
LVESVI	48.1±25.00	31.7±17.4	<0.001
LVEDV (mL)	132.1±56.1	90.3±31.3	<0.001
LVEDVI	78.7±36.00	52.8±20.6	<0.001
WMSI	2.1±0.50	1.6±0.4	<0.001
LAD (cm)	4.2±0.60	3.7±0.5	<0.001
MVEV (m/s)	0.9±0.3	0.7±0.2	0.002
MVAV (m/s)	0.8±0.2	0.7±0.2	0.13
MVDCT (ms)	136.7±70.4	195.3±100.2	0.001
MV E/A	49.9±97.9	117.7±115.8	0.003
MV annulus size-4chamber (cm)	3.0±0.4	2.9±0.3	0.03
MV annulus size-4chamber index	1.8±0.3	1.7±0.2	0.02
MV annulus size-PSLX (cm)	2.9±0.4	2.6±0.4	0.001
MV annulus size-PSLX index	1.7±0.4	1.5±0.3	0.004
MV annulus excursion (cm)	1.2±0.8	2.8±2.3	0.001
Inter-papillary muscle distance-diastolic (mm)	2.1±0.5	2.0±0.4	0.11
Inter-papillary muscle distance-systolic (mm)	1.5±0.5	1.3±1.2	0.005
Inter-papillary muscle distance-ratio (%)	32.8±12.8	42.4±12.0	<0.001
RVDD (cm)	3.1±0.4	3.0±0.5	0.23
RVDD index	1.9±0.3	1.7±0.3	0.09
TV excursion (cm)	1.7±0.4	1.9±0.2	0.002
PAP (mm Hg)	43.2±19.7	29.7±9.7	0.01
MV regurgitation volume (mL)	28.7±16.00	-	-
MV tenting area (cm^2^)	1.9±0.8	-	-
MV tethering depth (cm)	1.3±1.2	-	-
EROA (cm^2^)	0.2±0.1	-	-
Vena contracta width (mm)	2.6±1.4	-	-
S lateral (cm/s)	4.90±0.24	8.91±0.25	<0.001
S septal (cm/s)	6.98±0.18	4.98±0.25	<0.001
A' lateral (cm/s)	5.90±0.25	10.91±0.18	<0.001
A' septal (cm/s)	6.98±0.30	6.96±0.40	0.88
E' lateral (cm/s)	4.14±0.44	8.89±0.42	<0.001
E' septal (cm/s)	5.92±0.41	3.97±0.42	<0.001
E/E’	18.00±1.35	10.96±1.73	<0.001

Mechanism of IMR in case group was mono leaflet tethering in 44 cases, both leaflets tethering in 6 cases and ring dilatation in 31 cases. MR jet origin-direction was medial commisure-posterior in 33 (66%), lateral commisure-anterior in 11 (22%) and both commisure-central direction in 6 (12%).

Case group compared to control group had significantly higher LV (96% vs. 84%, *P *= 0.04) and RV diastolic dysfunction (32% vs. 6%, *P *= 0.003).

## Discussion

We observed that cases with IMR are more female, older and smoker. In the literature, cases with moderate to severe IMR were older,^8^ with female dominance.^9^

The risk of heart failure increases in moderate and severe IMR. In our study, these patients had significantly higher Killip class and lower LVEF. Fazlinezhad and colleagues^10^ observed higher rate of LVEF <35% in the patients with IMR. Lower LVEF was also reported in the cases with moderate to severe IMR.^9^

Different factors have been reported in the occurrence of IMR; these include LV remodeling, papillary muscle displacement, LV contractile dysfunction, structural changes of the valvular ring, and ventricular electro-mechanical dyssynchrony.^11,12^ In our cases with moderate to severe IMR, the mechanism was mono leaflet tethering in 44 cases, both leaflets tethering in 6 cases and ring dilatation in 31 cases. MR jet origin-direction was medial commisure-posterior in 33, lateral commisure-anterior in 11 and both commisure-central direction in 6.

In our study, case group also had significantly abnormal echocardiographic and tissue Doppler findings as well as higher LV and RV diastolic dysfunction.

Effective regurgitant orifice area (EROA), calculated with PISA and Doppler methods, is a criterion of MR severity independent of hemodynamic state and also an independent predictor of prognosis. EROA ≥20 mm^2^ is considered as a cut-off for moderate MR and undesirable outcome. Vena contracta dimensions reflect the severity of regurgitation.^14^ Agricola and colleagues^13^ reported tenting area of 1 cm^2^ and coaptation depth of 5.5 mm as normal values. They reported these two parameters to be correlated with MR severity and left ventricle dysfunction. In our study, EROA was 0.2 cm^2^. Tenting area and tenting depth in IMR was 1.9 cm^2^ and 10 mm, indicative of Severity of MR.

Severity of MR could be measured by different methods including distal jet area, vena contracta, proximal isovelocity surface area (PISA) method, volumetric, continuous wave Doppler pattern, tenting area and tethering depth (height). Previous studies have indicated that tenting area ≥1.5 cm^2^ & tethering depth ≥1 cm are indicative of severe MR,^15,16^ and we reached the similar results. On the other hand, an elevated RV systolic pressure, LA and LV enlargement, an increased E wave velocity are also supportive of significant MR. The most scientific and accurate method is integration of the sum of all these measures. In fact, according to the sum of the available data, MR was more sever in case group.

Different echocardiographic findings are reported in cases with moderate to severe IMR. It is reported that moderate and severe MR in acute MI is related to increased left ventricular diastolic dimensions.^8^ Fazlinezhad and colleagues^10^ reported higher grade of diastolic dysfunction, end-diastolic LV pressure in IMR cases. They also had higher Systolic Pulmonary Artery Pressure (SPAP) which was related directly to the severity of MR. MacHaalany et al^9^ also showed higher prevalence of PAP, LVESD and LA size in moderate to severe IMR. However, in the Lamas et al study,^15^ the patients with IMR and those without MR had similar LV filling pressures.

In our study, the mean value of LVEDV, LVEDVI, LVESV and LVESVI are mild to moderately enlarged which are predictable considering the local remodeling as a consequence of RWMA and papillary muscle displacement in inferior MI. Observing monoleaflet tethering in most patients in case group is also indicative of local remodeling, as in case of global remodeling we observe bileaflet tethering. Along with these findings, having Ant MI in 20% of cases could be effective in the increase in the volumes.

It is reported that the occurrence and severity of the IMR is affected by the location of MI; the mechanism producing IMR is different in anterior compared with inferior STEMI. Although LV remodeling and global LV dysfunction is greater in anterior MI, IMR is more severe in inferior STEMI due to the increased tethering force of the posteromedial papillary muscle near the site of the infarction.^17,18^ Also, IMR is more likely to occur in the inferior MI that anterior MI.^19^ The higher incidence of IMR in inferior MI is reported in previous studies.^8,17^

Similarly in our study, we observed that inferolateral MI was higher in moderate to severe IMR. This group also received less streptokinase. It is reported previously that primary PCI lowers the incidence of MR in STEMI patients,^20^ and receiving thrombolytic therapy or late PCI may increase the rate of IMR.

## Study limitations

We only included 50 cases with IMR and 50 cases without IMR which could cause selection bias. Most patients underwent PCI in the few first days after MI and before we could evaluate them by echocardiography; so they were excluded. The study sample was also low and could limit our analysis.

## Conclusion

IMR is common after AMI, especially in cases with inferior MI. The echocardiographic findings are indicative of left ventricular remodeling and abnormality of mitral valve apparatus.

## Ethical approval

All patients gave informed written consent and the ethics committee of Tabriz University of Medical Sciences approved the study protocol.

## Competing interests

None.

## Funding

This study was supported by a grant from Cardiovascular Research Center, Tabriz University of Medical Sciences, Tabriz, Iran. Authors have no other funding support to disclose.
